# Editorial: Promoting patient and caregiver engagement in chronic disease management

**DOI:** 10.3389/fpsyg.2023.1324935

**Published:** 2023-11-01

**Authors:** Giada Rapelli, Chiara Acquati, Emanuele Maria Giusti, Silvia Donato

**Affiliations:** ^1^Department of Medicine and Surgery, University of Parma, Parma, Italy; ^2^Graduate College of Social Work, University of Houston, Houston, TX, United States; ^3^Department of Medicine and Surgery, EPIMED Research Center, University of Insubria, Varese, Italy; ^4^Department of Psychology, Catholic University of the Sacred Heart, Milan, Italy

**Keywords:** self-management, chronic disease, health outcome, dyadic/individual processes, caregiving

A chronic illness exerts a profound impact on an individual's physical and mental wellbeing, while also influencing their identities, familial relationships, and social responsibilities. Managing a chronic condition is a lifelong endeavor that demands substantial lifestyle adjustments, strict adherence to medication and treatment regimens, and the implementation of preventive measures. Informal caregivers, typically partners, spouses, family members, and friends, play a pivotal role in this experience: they are tasked with a range of complex and demanding responsibilities, such as responding to emergencies, mediating disagreements between patients and physicians, participating in treatment decisions, and addressing patients' emotional and practical needs. Promoting and supporting both patient and caregiver engagement is therefore vital for effective management of the disease.

This Research Topic (RT) delves into the complex interplay between individuals facing chronic health conditions and their caregivers. The contributions included emphasize the intricate dynamics of patient-caregiver relationships and the interdependence between their actions and outcomes, encompass diverse populations such as patients and caregivers facing cardiac diseases, multiple sclerosis, amyotrophic lateral sclerosis, hemodialysis, and multimorbidity. The studies presented shed light on the theme of engagement by delving into the topic of caregiver burden and by presenting psychological interventions health education programs, and self-care programs aimed at increasing engagement.

Three of the studies included in the present RT (Golan and Vilchinsky; Rapelli, Giusti, Tarquinio, et al.; Rapelli, Giusti, Donato, et al.) explored the relationship between patient and caregiver wellbeing and different aspects of cardiac disease management. The study by Rapelli, Giusti, Tarquinio, et al. offered a scoping review of the existing psychological couple-oriented interventions conducted for patients with heart disease and their partners. The review collected 11 studies with heterogeneous designs, theoretical backgrounds, types of intervention, personnel involved, intervention formats (group or individual, phone or in person), number of sessions, and duration. Results indicated high variability among couple-based interventions, lack of adequate details regarding the training of professionals delivering them, the contents of the interventions, and the theoretical models informing these programs. Interventions for patients with cardiovascular disease and their partners were found to moderately improve psychosocial outcomes. Furthermore, in most studies, only the patients' psychological strategies and outcomes were considered. Current contributions also revealed the stress experienced when a transplant or the implantation of an external device such as the Left Ventricular Assist Device (LVAD) is needed. The qualitative study by Rapelli, Giusti, Donato, et al. utilizes a phenomenological hermeneutic approach to understand the experience of six patient-caregiver dyads undergoing cardiac rehabilitation Four were the main themes: *Being between life and death, Being human with a heart of steel, Sharing is caring (and a burden), and Being small and passive*. Finally, Golan and Vilchinsky reflect on the necessity of applying a dyadic approach in the context of Left Ventricular Assist Device transplantation to enriching the understanding of patients' coping with this disease.

One study (Mousaei et al.) considered the issue of care burden in informal caregivers of patients with multiple sclerosis (MS), given the risk of impaired quality of life, physical, and mental wellbeing of this population. The authors illustrate the Family-Centered Empowerment Model (Intervention Group; IG) and compared it with TAU (Control Group; CG), showing that the intervention proposed does not have a favorable effect in reducing the care burden among caregivers of patients with MS. Another article (Huang et al.) contributed to the literature about caregiver-focused interventions. The authors aimed to evaluate the effectiveness of a health education program based on the “Timing it Right” framework (IG) on the caregivers' care ability, emotions, and health-related quality of life in the context of haemodialysis. However, the intervention had no significant effect in alleviating care burden. Similarly, the study protocol by Gomes de Souza e Silva et al. aims to investigate the effects of a self-care education program via telerehabilitation on the burden and quality of life of caregivers of individuals with Amyotrophic Lateral Sclerosis. This program has the potential to be widely disseminated among health professionals, increasing remote monitoring of individuals with difficulty performing practical care activities.

Care coordination and distress increase in case of multimorbidity (i.e., being diagnosed with at least two chronic conditions), the physical, psychosocial, and relational changes and aspects of threats elicited by multimorbidity also provoke significant emotional responses. The qualitative study by Horn et al. explored the types of emotional co-regulation that couples facing multimorbidity express. Three key themes emerged from the interviews. First, we-ness (conceiving of the pair as a unit) and overlapping cognitive assessments of the circumstance (ranging from fighting spirit to fatalism) emerged as higher-order factors from the quotations. Second, relationship-related tactics, such as striking a balance between autonomy and equity in the couple, were frequently highlighted. Third, some couples discussed the advantages of individual strategies that focus on developing partners' resources outside of their romantic partnerships (such as creating relationships with their grandchildren or spending time in nature).

This Research Topic underscores the significance of recognizing chronic illness management as a collaborative experience shared by patients and informal caregivers. The studies presented shed light on the complexities of patient-caregiver relationships, offering insights into the effectiveness of interventions and the evolving needs of those navigating chronic illnesses. By adopting a dyadic perspective, healthcare professionals can better support individuals facing chronic illnesses and their caregivers and tailor care approaches to their needs. Future research should continue to explore dynamics within these partnerships and develop tailored interventions that address the multifaceted challenges they encounter.

## Author contributions

GR: Writing – original draft. CA: Writing – original draft. EG: Writing – original draft. SD: Writing – original draft.

